# Automatic Detection of Atrial Fibrillation from Single-Lead ECG Using Deep Learning of the Cardiac Cycle

**DOI:** 10.34133/2022/9813062

**Published:** 2022-04-12

**Authors:** Alina Dubatovka, Joachim M. Buhmann

**Affiliations:** Department of Computer Science, ETH Zurich, Zurich, Switzerland

## Abstract

*Objective and Impact Statement*. Atrial fibrillation (AF) is a serious medical condition that requires effective and timely treatment to prevent stroke. We explore deep neural networks (DNNs) for learning cardiac cycles and reliably detecting AF from single-lead electrocardiogram (ECG) signals. *Introduction*. Electrocardiograms are widely used for diagnosis of various cardiac dysfunctions including AF. The huge amount of collected ECGs and recent algorithmic advances to process time-series data with DNNs substantially improve the accuracy of the AF diagnosis. DNNs, however, are often designed as general purpose black-box models and lack interpretability of their decisions. *Methods*. We design a three-step pipeline for AF detection from ECGs. First, a recording is split into a sequence of individual heartbeats based on R-peak detection. Individual heartbeats are then encoded using a DNN that extracts interpretable features of a heartbeat by disentangling the duration of a heartbeat from its shape. Second, the sequence of heartbeat codes is passed to a DNN to combine a signal-level representation capturing heart rhythm. Third, the signal representations are passed to a DNN for detecting AF. *Results*. Our approach demonstrates a superior performance to existing ECG analysis methods on AF detection. Additionally, the method provides interpretations of the features extracted from heartbeats by DNNs and enables cardiologists to study ECGs in terms of the shapes of individual heartbeats and rhythm of the whole signals. *Conclusion*. By considering ECGs on two levels and employing DNNs for modelling of cardiac cycles, this work presents a method for reliable detection of AF from single-lead ECGs.

## 1. Introduction

Cardiac arrhythmias characterise a group of various heart conditions where heart rhythms do not follow a normal healthy sinus pattern. Atrial fibrillation (AF) arises among the most common arrhythmias occurring in 1–2% of the general population [1], with an age-dependent population prevalence of 2.3–3.4% [2]. The incidence rates of AF increased significantly over the past 15 years [3]. Due to the increased mortality associated with arrhythmias, patients critically depend on a timely diagnosis [2, 4] accompanied by medication or surgical interventions. Electrocardiogram (ECG) is a major tool for diagnosis of cardiovascular diseases [5], including cardiac arrhythmias [4], because of its availability and cost. Today, mobile recorders enable patients to record ECGs remotely using single-lead devices. However, these devices produce recordings with lower signal-to-noise ratio and lower frequency than standard clinical monitors. Also, the length of mobile recordings is not standardised and may vary considerably. Therefore, reliable detection of AF based on single-lead ECG remains a challenging and error-prone diagnostic task. Moreover, the broad taxonomy of heart rhythms and occasional occurrences of arrhythmic episodes renders AF hard to distinguish from other forms of arrhythmias.

Deep neural networks (DNN) [6] showed significant progress in solving various sequence classification tasks including speech recognition [7], machine translation [8], video recognition [9], strategy games like Atari, chess, and Go [10], and protein structure prediction [11]. In the context of medicine, DNNs were successfully used for dermatology [12], diabetes prediction [13], arrhythmia classification [14], and other applications [15].

However, existing DNN models are rather generic and do not utilize unique features of analyzed signals. For instance, most of the DNN algorithms for arrhythmia detection are based on convolutional neural networks (CNN) [16–20] which were adapted from computer vision tasks and not designed for ECG signals. Thus, DNNs used for analyzing ECG signal do not take into account its periodic nature and process the entire signal instead of focusing on individual heartbeats. Yet, some properties of heartbeats such as heart rate variability described as the standard deviation of the length of heart cycles are known to be descriptive features for arrhythmia detection [21–25].

As a second important issue of ML methods in medicine, DNNs most often do not support a functional understanding of their decision-making process. Due to their black-box nature and huge number of parameters, it is challenging to understand what caused a certain output of the model [16]. For the medical applications, however, the reasoning behind algorithmic decisions especially when based on patient data is indispensable for medical experts to judge the validity of the diagnostic process.

In [26], the authors propose the DeepHeartBeat (DHB) framework, an autoencoder-based model, to learn an interpretable low-dimensional representation of echocardiogram videos (ECHOs) and electrocardiograms (ECGs) in an unsupervised way. The DeepHeartBeat approach explicitly models the periodic nature of the cardiac cycle and captures periodic features of the data together with the frequency of the rhythm. However, their work focuses on short echocardiogram videos (ECHOs) and assumes that the frequency of the cardiac cycle stays constant for the entire signal. This assumption does not hold for electrocardiograms recorded from arrhythmic patients due to longer duration of the ECG recordings and irregular heart rhythm of the arrhythmic patients.

In this paper, we investigate the applicability of DeepHeartBeat to ECG signal for diagnosis of atrial fibrillation and other types of cardiac arrhythmias. Because the DeepHeartBeat framework is designed to work with relatively short signals, we discuss different strategies for extracting subsequences from the original signal for encoding. Additionally, we propose approaches for aggregating the encoded subsequences into a single representation of the signal for performing downstream tasks. Finally, we describe and compare two simplified versions of the DeepHeartBeat method which rely on the preprocessing of ECGs and preceding splitting procedure to reduce the number of features learnt by the model.

The rest of the paper is organized as follows. Section 1 contains the introduction, Section 2 reviews the related work, Section 3 describes our approach and experimental pipeline, Section 4 reports the results, and Section 5 discusses the limitation of the proposed approach.

## 2. Related Work

In this section, we review some of the state-of-the-art approaches for AF detection for ECG signals. The related work can be divided into two categories: traditional approaches (Section 2.1), including machine learning solutions, and deep learning models (Section 2.2). The main differences between these categories arise from the richness of the model class; i.e., deep learning algorithms learn features from data automatically while the traditional approaches rely on hand-crafted and predefined features.

### 2.1. Traditional Methods

Traditional approaches for automatic atrial fibrillation detection usually rely on manually crafted features extracted as a first step of the detection pipeline. These features mostly resemble two main characteristics of AF ECG signals: absence of P-waves and irregularity of R-R intervals (RRIs). The absence of a P-wave as well as other features proved to be a fragile, unreliable preprocessing of the ECG data in the presence of noise, since these methods depend on robust QRS-complex extraction. Although Asgari et al. [27] proposed to apply a wavelet representation to extract peak-to-average power ratio and log-energy entropy to eliminate the detection of P-wave and R-peak, they still rely on hand-crafted feature design. Therefore, RRI-based methods still serve as a strong baseline in this category.

For instance, Islam et al. [23] proposed a normalization procedure to discard the effect of ectopic heartbeats of the AF signals before computing normalized entropy as a measure of irregularity of heartbeat duration in a fixed-length window. However, similar to many RRI-based approaches, it requires long recordings (30-70 heartbeats) to identify AF. In [24], the authors propose a linear transformation of a window of RRI tachogram based on neighbourhood component analysis followed by a naïve Bayesian classification of the transformed signal to achieve state-of-the-art performance on the MIT-BIH Arrhythmia Database [28] when considering shorter recording of only 15 beats.

### 2.2. Deep Learning Methods

Deep learning (DL) methods distinguish themselves by their ability to learn task-specific features from available data contrary to traditional methods that depend on the manually crafted features. Similarly to the conventional machine learning approaches described above, DL algorithms can be applied to both RRI tachograms and raw ECG signals. For example, Andersen et al. [25] apply deep learning (ensemble of CNN and RNN models) to detect AF from an input of 30 consecutive RRI tachograms. Most of the approaches, however, apply deep neural networks (DNN) to raw ECG signal directly to learn feature mappings. Convolutional neural networks (CNN) have shown convincing capability in feature extraction for computer vision tasks; therefore, many researchers adapted CNNs to solve AF detection task. Fan et al. [20] explore multiscale fusion of deep CNN networks (MS-CNN) to detect AF signals based on single-lead ECG recordings from the Physionet Challenge database [29]. An end-to-end deep visual network called ECGNET [30] automatically detects AF in very short ECG recordings (around 5 s). The approach was tested using the signals from the MIT-BIH Atrial Fibrillation Database [31]. However, the authors excluded other rhythms in the data that are also present in the database and considered a simple dichotomy of distinguishing between AF and normal sinus rhythm (NSR).

Hannun et al. [14] employed a CNN algorithm to achieve state-of-the-art performance in classifying ECG beats into fourteen different classes. However, they utilized a large amount of privately collected, not publicly accessible data for training their model.

## 3. Materials and Methods

### 3.1. Experimental and Technical Design

**Figure 1:**
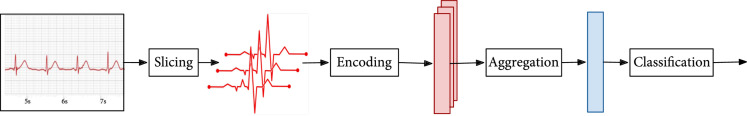
Processing pipeline.

The overall design of our experimental pipeline consists of four main steps as depicted in Figure 1.

(i) *Slicing* of the original signal into (possibly overlapping) subsequences of variable lengths covering the signal (Section 3.2)
(ii) *Encoding* the subsequences to obtain a low-dimensional representation of each subsequence (Section 3.3)
(iii) *Aggregation* of all the representations of subsequences extracted from the same signal in order to obtain a single representation of the entire signal (Section 3.4)
(iv) *Classification* of the computed representations to perform diagnosis of cardiac dysfunctions (Section 3.5)

### 3.2. Slicing

The original DHB framework only allows us to encode relatively short sequences of few heartbeats and cannot capture changes in heart rhythm. Since such rhythmic variations are essential for detecting arrhythmias, we have to design a strategy how to represent a long ECG recording as a sequence of shorter subsequences extracted from that recording. Below, we describe three main strategies for such slicing: random slicing, R-peak aligned slicing, and heartbeat extraction.

#### 3.2.1. Random Slicing

Subsequences are extracted from the original ECG recording starting at randomly selected time points with random duration between 1.5 and 4.0 seconds. This approach to sequence slicing generates as many slices from a recording as needed for training, thereby empowering the learning algorithm to train more sophisticated models with larger number of parameters. Furthermore, random slicing provides an additional data augmentation. However, there does not exist any guarantee that the whole recording will be represented by the learning algorithm. In particular, important pieces of the ECG signal might get overlooked during training and might not be captured by the latent representation of deep learning. Additionally, the slices contain different numbers of heartbeats and start at different points of the cardiac cycle which might render training of DNN complicated as the model would need to accommodate for that.

#### 3.2.2. R-Peak Aligned Slicing

The ECG signal expresses a clear periodicity of heartbeats. Therefore, data analysts can extract subsequences starting from a well-defined time point of the cycle and can thereby align the subsequences so that the model automatically learns shift invariant information. We ensure such an alignment by extracting positions of R-peaks using Pan-Tompkin’s algorithm [32] and by always starting subsequences from one of the R-peaks of the signal. The length of the extracted subsequences may be chosen randomly as for the random slicing approach above.

#### 3.2.3. Heartbeat Extraction

Taking this slicing strategy a step further, we can ensure that extracted subsequences not only are aligned but also contain the same number of heart cycles. This standardization is achieved by extracting the signal between two consecutive R-peaks of the recording such that every subsequence consists of exactly one heart cycle. Thereby, we extract the heart rate directly from the ECG signal rather than learning it from nonaligned ECG slices.

### 3.3. Encoding

After an ECG recording is split into multiple slices, we need to extract relevant features of each subsequence. The features should capture the heart cycle and heartbeat shape since both pieces of information are relevant for the diagnosis of cardiac diseases. In extension of the original DeepHeartBeat method introduced in [26], we modify this framework by exploiting some advantages of R-peak aligned slicing and heartbeat extraction strategies as described in Section 3.2.

#### 3.3.1. DeepHeartBeat

DeepHeartBeat (DHB) is an autoencoder-based framework for learning cyclic latent trajectories of periodic sequences [26]. Given a signal ${\left(s_{j},t_{j}\right)}_{j=1}^{n}$, where $s_{j}$ corresponds to a measurement at time $t_{j}$, e.g., for ECGs, it is a voltage value at $t_{j}$, DHB maps the signal into a vector of trajectory parameters $\varphi_{i}^{\text{DHB}}=\left(f_{i},\tau_{i},b_{i}^{\left(3\right)},\cdots ,b_{i}^{\left(d\right)}\right)\in {\mathbb{R}}^{d}$ using a deep neural network encoder. The frequency parameter $f_{i}>0$ corresponds to the number of heart cycles per time unit, and the shift parameter $\tau_{i}$ accommodates for the fact that subsequences start at different moments within the heart cycle. The $b_{i}$-parameters capture the shape of the input signal, e.g., the shape of a heartbeat. The parameter vector $\varphi_{i}$ induces a cyclic trajectory $\ell_{i}\left(t\right)$ over time in a lower dimensional latent space ${\mathbb{R}}^{d}$ as described in

(1)ℓiDHBt=cos2πfit−τie1+sin2πfit−τie2+∑j=3dbijej,

where $\left(e_{1},\cdots ,e_{d}\right)$ is the canonical basis of ${\mathbb{R}}^{d}$ in which the sequences are embedded. To integrate prior knowledge about the periodicity of sequence i, the reconstruction ${\left({\hat{s}}_{j},t_{j}\right)}_{j=1}^{n}$ of the input sequence is computed as a mapping from the point of the latent trajectory $\ell_{i}\left(t\right)$. In other words, ${\hat{s}}_{j}=f_{D}\left(\ell_{i}\left(t_{j}\right)\right)$, where $f_{D}$ is a decoder function represented by a neural network.

#### 3.3.2. Pace-DeepHeartBeat

Because we can extract R-peaks of heartbeats from the ECG signal, we can align extracted slices by forcing them to always start with a R-peak. In this case, the shift parameter τ of the original DeepHeartBeat parameterisation described above becomes obsolete because all input subsequences start from the same point of the cycle. We therefore propose a simplified version of DeepHeartBeat without the shift parameter τ, called Pace-DeepHeartBeat (P-DHB). Equation (2) outlines the corresponding trajectory parameters and latent trajectory. Please note that although the embedding $\varphi_{i}$ is now represented by $\left(d-1\right)$ dimensions as we omit the $\tau_{i}$ component, the resulting trajectory $\ell_{i}^{\text{P}‐\text{DHB}}\left(t\right)$ still evolves in a d-dimensional space.

(2)φiP‐DHB=fi,bi3,⋯,bid,ℓiP‐DHBt=cos2πfite1+sin2πfite2+∑j=3dbijej.

#### 3.3.3. Shape-DeepHeartBeat

In order to simplify the original DeepHeartBeat parameterisation even further, we remove the pace parameter $f_{i}$, too, and only include the shape parameters $b_{i}$ into the parameterisation $\varphi_{i}$. The resulting signal parameterisation and the latent trajectory, hence, assume the form of equation (3). By omitting the first two components $f_{i},\tau_{i}$, the embedding $\varphi_{i}$ has $\left(d-2\right)$ dimensions, but the latent trajectory $\ell_{i}^{\text{S}‐\text{DHB}}\left(t\right)$ still evolves in d dimensions. We call this version Shape-DeepHeartBeat (S-DHB).

(3)φiS‐DHB=bi3,⋯,bid,ℓiS‐DHBt=cos2πte1+sin2πte2+∑j=3dbijej.

### 3.4. Aggregation

Since all the methods described in the previous section are based on DeepHeartBeat, they are not suitable for processing long signals. From a technical perspective, the sequential encoder of DeepHeartBeat cannot handle long input sequences. Adopting a conceptual viewpoint, the DeepHeartBeat parameterisation implies a fixed heart rate for the entire signal which is a rather unrealistic assumption for arrhythmic patients. To overcome these issues, we propose to split a long input signal into short subsequences such as individual heartbeats or short signal slices and apply DHB to encode them. Therefore, a means to combine the learned representations of the subsequences into a single embedding for the entire signal is required. For example, if ${\left\{\varphi_{ik}\right\}}_{k=1}^{n_{i}}$ are $n_{i}$ embeddings of heartbeats or slices extracted from the recording of patient i, then the embedding vector of the recording can be expressed as $\varphi_{i}=f\left(\varphi_{i1}\cdots \varphi_{in_{i}}\right)$. We denote the function f as an aggregation function.

#### 3.4.1. Averaging

As a baseline method to obtain a fixed-size vector representation for a signal of variable length, we propose averaging of the learnt representations of all slices from the same signal. Namely, for the subject i, we define its representation $\varphi_{i}=\left(f_{i},\tau_{i},b_{i}^{\left(3\right)},\cdots ,b_{i}^{\left(d\right)}\right)$ based on the representations ${\left\{\varphi_{ik}\right\}}_{k=1}^{n_{i}}$ of the subsequences as follows ($n_{i}$ is the number of subsequences representing the i-th signal):

(4)φi=1ni∑k=1niφik.

#### 3.4.2. Recurrent Neural Network

While aggregation of the embedding via averaging generates a single representation of the signal from the embeddings of subsequences, it suffers a major information loss as a drawback. Averaging discards information about the order of the input subsequences and the variance of the features learnt from different heartbeats. This information, however, is proven to be relevant for detection of many cardiovascular diseases associated with heart rhythm abnormalities [22].

To take this sequence ordering into account, we consider a more sophisticated aggregation function represented by a recurrent neural network (RNN) [6]. RNNs process each element of an input sequence in order, and the output of a step is dependent on the previous computation. Therefore, the network has a memory to accumulate information about previously seen elements, dynamics of data, and the state of computations until the current step. In particular, we use a Long-Short-Term Memory (LSTM) cell [33] to process sequences of the embeddings ${\left\{\varphi_{i}\right\}}_{k=1}^{n_{i}}$ as described in equation (5). Table S2 in Supplementary Materials outlines the architecture of the RNN which we used in our experiments.

(5)φi=RNNφi1,⋯,φini.

### 3.5. Classification

For performing a downstream task, the aggregated signal representations $\varphi_{i}$ are passed to a task-specific DNN. For a classification task, a fully connected DNN outputs probabilities that recording i belongs to each of the diagnostic classes. In our case, there are four classes representing normal sinus rhythm, atrial fibrillation, an alternative rhythm, and recordings that are too noisy to classify. We provide more information about the classification datasets in Section 4. Table S3 in Supplementary Materials summarizes the DNN architecture of the classifier used for our experiments.

### 3.6. Statistical Analysis

We statistically evaluate our approach in two different ways: first, we reconstruct the original signal based on the learnt sequence representation and, thereby, assess the quality of this reconstruction. The reconstruction quality is quantified by the root-mean-square error (RMSE) between the original and reconstructed subsequences outlined in

(6)RMSEsj,s^j=1nj∑k=1njsjk−s∧jk2,

where $s_{j}$ is an input subsequence of length $n_{j}$ and ${\hat{s}}_{j}$ is the DHB reconstruction of $s_{j}$. We compare different versions of DeepHeartBeat described in Section 3.3 as well as different dimensionalities of latent space d.

Then, we utilize the aggregated signal representations for performing a downstream classification task, namely, atrial fibrillation diagnosis using three different datasets: the PhysioNet/Computing in Cardiology Challenge 2017 [29], MIT-BIH Atrial Fibrillation Database [31], and MIT-BIH Arrhythmia Database [28]. In particular, we evaluate how well the models perform a binary classification between AF and non-AF rhythms (normal rhythm, noise, and other abnormal heart rhythms) by reporting the F-1 score, sensitivity, specificity, PPV, area under the ROC curve (AUC), and accuracy. The F1-score, sensitivity, specificity, PPV, and accuracy were calculated at the binary decision threshold of 0.5. The F1-score is the harmonic mean of the PPV and sensitivity. It scores models in the range of 0 to 1, and it ranks models that maximize both PPV and sensitivity simultaneously higher than models that boost only one of them. In the presence of class imbalance, the F1-score provides complementary information to the AUC score [34].

All the classification statistics described above are provided as a mean and standard deviation over 10-fold leave-one-out cross validation. For this purpose, each dataset is divided into 10 equal-sized folds. We then trained 10 models each time using one fold for testing and the remaining nine folds for training the models and report mean test performance of these 10 models alongside the standard deviation. The cross-validation procedure was employed to estimate robustness of the models with respect to data variability and not for parameter tuning; we kept all hyperparameters fixed and identical for all models.

## 4. Results

For our experiments, we use data from the PhysioNet/Computing in CardiologyChallenge 2017 [29, 35], MIT-BIH Atrial Fibrillation Database [31, 35], and MIT-BIH Arrhythmia Database [28, 35]. The PhysioNet/CinC Challenge 2017 (Physionet Challenge) dataset consists of 8528 single-lead ECG recordings between 9 and 61 seconds in length. The recordings were acquired using AliveCore’s single-channel ECG device and stored with 300 Hz frequency and ±5 mV dynamic range. No preprocessing, filtering, or normalization was applied to the ECG signals. Each ECG signal is labeled as one of four classes: normal sinus rhythm (NSR), atrial fibrillation (AF), an alternative rhythm, or being too noisy to be classified. Table S1 from Supplementary Materials provides statistics about the length and class label distributions of the training data.

**Figure 2:**
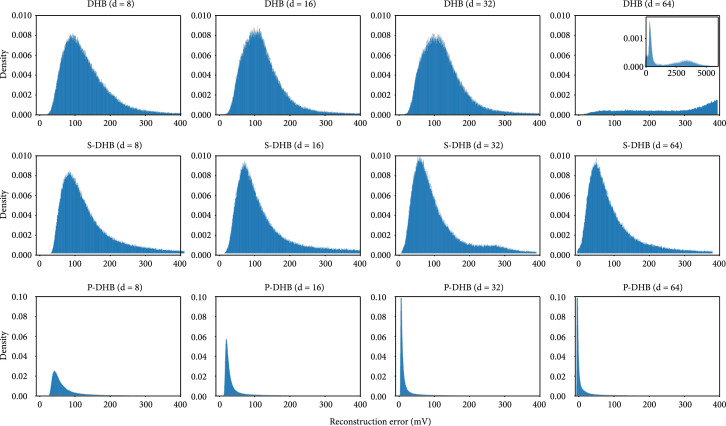
Histograms of root-mean-square error (RMSE) for DeepHeartBeat, Shape-DeepHeartBeat, and Pace-DeepHeartBeat (rows) with different numbers of latent dimensions d (columns). The error histogram for Pace-DeepHeartBeat is significantly sharper distributed at low values which requires a ten times larger scale in the plots. The breakdown of DeepHeartBeat for d=64 is seen on the broad histogram at the large error values.

As outlined in Section 3, we first evaluated the proposed encoders in terms of the quality of signal reconstruction; the reconstruction errors have been estimated on the basis of approximately 318000 heartbeat events from the Physionet Challenge dataset. Table 1 summarizes the reconstruction errors for different versions of DeepHeartBeat and different numbers of dimensions of the latent space d. Figure 2 depicts histograms of the reconstruction error for each of the configurations. Pace-DeepHeartBeat produces significantly better signal reconstructions than DeepHeartBeat and Shape-DeepHeartBeat for all numbers of the latent space dimensions d. Moreover, the reconstruction quality of Shape-DeepHeartBeat degrades more gracefully than that of DeepHeartBeat that shows a complete failure in signal reconstruction for 64 or more latent parameters.

**Table 1:** Reconstruction error of different encoders and different numbers of dimensions d of the latent trajectories based on approximately 318000 heartbeat events. Reconstruction error is measured as root-mean-square error (RMSE, equation (6)) in mV. Average RMSE over all training sequences is presented with 95% confidence interval, assuming an exponential distribution of the error values.

Encoder	d=8	d=16	d=32	d=64
DHB	136.1 [11.2–480.9]	129.2 [9.1–460.6]	135.7 [8.9–485.5]	1927.4 [53.0–7098.1]
S-DHB	140.4 [11.9–494.8]	137.5 [7.7–495.5]	123.1 [8.6–438.9]	161.3 [10.3–577.7]
P-DHB	54.4 [5.7–188.7]	29.8 [3.8–101.5]	19.5 [3.0–65.1]	18.2 [3.2–59.4]

**Figure 3:**
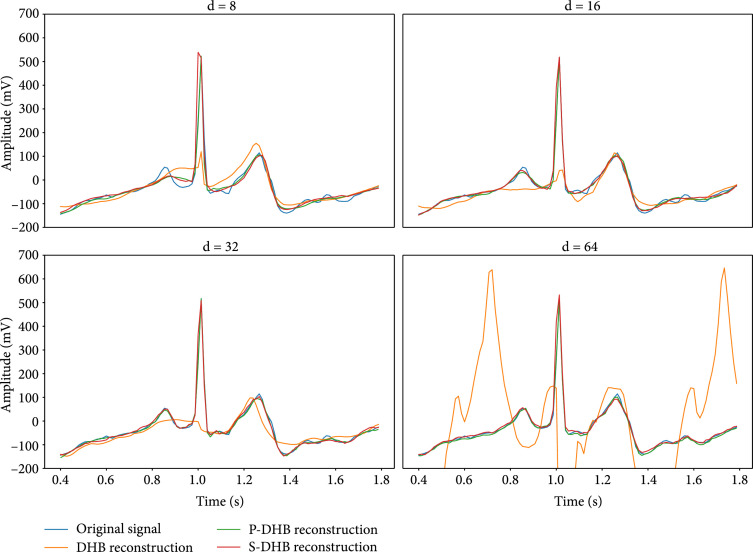
Reconstruction of a single heartbeat by DeepHeartBeat, Pace-DeepHeartBeat, and Shape-DeepHeartBeat with different numbers of latent dimensions d.

Figure 3 depicts an example of a heartbeat reconstructed by DeepHeartBeat, Pace-DeepHeartBeat, and Shape-DeepHeartBeat encoders with different numbers of latent space dimensions d. Pace-DeepHeartBeat and Shape-DeepHeartBeat produce more accurate reconstructions of heartbeats than the original method DeepHeartBeat since they are tailored for heartbeats and trained on R-peak aligned samples. However, 8 dimensions are not enough for Pace-DeepHeartBeat to encode all signal features necessary to reconstruct all the ECG wave components accurately. With 16 dimensions, the quality of the reconstruction improves significantly. In contrast, the reconstruction produced by DeepHeartBeat with 64 latent dimensions fits the original signal very poorly and shows many nonexistent waves as a result of overfitting. It also fails to learn the correct phase of a heart cycle, and therefore, the reconstructions miss the correct location of the R-peaks. Figure S1 from Supplementary Materials provides further examples of reconstructions of multiple heartbeats from the same patient.

**Figure 4:**
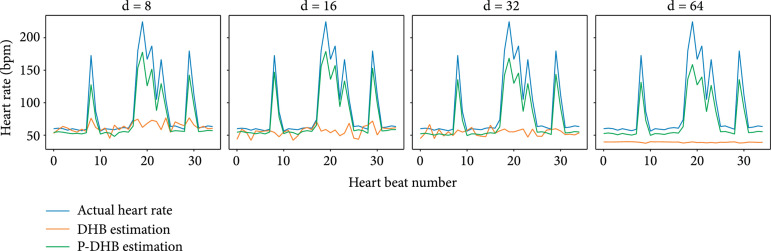
Estimation of the heart rate over the course of a signal produced by DeepHeartBeat and Pace-DeepHeartBeat with different numbers of latent dimensions d.

Additionally, we investigate how good the encoders estimate the heart rate of the input sequences. Since Shape-DeepHeartBeat does not explicitly model a heart cycle frequency, we only consider DeepHeartBeat and Pace-DeepHeartBeat for this experiment. Figure 4 presents the heart rate dynamics of a patient together with the heart rate estimations extracted by DeepHeartBeat and Pace-DeepHeartBeat. We can see that Pace-DeepHeartBeat makes a more accurate estimation of the heart rate than DeepHeartBeat, especially in cases of irregular occurrences of very short heartbeats. While DeepHeartBeat can capture average heart rate correctly, it uses only one frequency for the whole slice which in the case of the presence of irregular short heartbeats contains multiple heart cycles of different lengths. It is also worth noticing that DeepHeartBeat with 64 latent dimensions significantly underestimates even the average heart rate of a signal. This observation partially explains the low quality of the reconstruction presented in Figure 3 where the DHB with 64 latent dimensions predicts two R-peaks within one heartbeat cycle.

**Figure 5:**
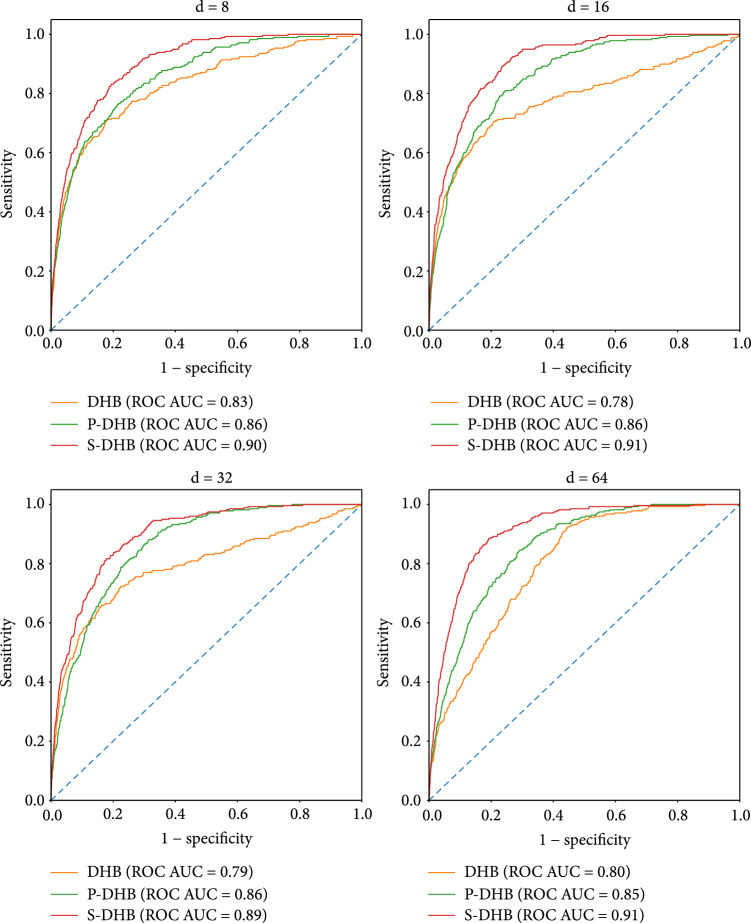
ROC curves for noise detection by DeepHeartBeat, Pace-DeepHeartBeat, and Shape-DeepHeartBeat with different numbers of latent dimensions d.

The Physionet Challenge dataset distinguishes itself by including noisy recordings as one of its challenges. Such recordings are demanding for classification due to their low signal-to-noise ratio; in practice, however, detection of noisy recordings is of great importance which emphasizes the realism of the Physionet Challenge. Noisy recordings amount to only 3.3% of the dataset, which converts their analysis into an anomaly detection problem. As noisy recordings do not show regular behaviour anymore, an autoencoder should face difficulty when reconstructing them. Since our model is designed to capture periodicity of the input signal and of typical heartbeat shape, we select the reconstruction quality as an informative criterion for detecting such noisy signals. To test this hypothesis, we encode and reconstruct every heartbeat from each ECG recording and then employ the average heartbeat reconstruction error of a signal as a predictor for the noise class which yields excellent results with an AUC score up to 0.91. Figure 5 presents ROC curves for DeepHeartBeat, Phase-DeepHeartBeat, and Shape-DeepHeartBeat with different numbers of latent dimensions d. In agreement with the results presented before, DeepHeartBeat performs significantly worse than Pace-DeepHeartBeat and Shape-DeepHeartBeat. Surprisingly, however, Shape-DeepHeartBeat shows the highest AUC for the noise prediction task score among the autoencoders despite having lower reconstruction quality than Pace-DeepHeartBeat. We attribute this robustness to the fact that the heartbeat extraction strategy described in Section 3.2 can capture information about noise because heartbeat extraction algorithms are sensitive to signal quality.

We then compare the performance of different autoencoders and aggregation strategies described in Section 3 on the downstream classification task of detection of atrial fibrillation (AF). Table 2 shows the performance of the proposed approaches in comparison with other works evaluated on the Physionet Challenge dataset. Our approach achieves 97% accuracy and significantly outperforms other approaches except for MS-CNN [20] where it performs on par. However, MS-CNN requires additional data balancing and augmentation strategies for training given a relatively small size of the dataset and high class imbalance. Additionally, Table S4 in Supplementary Materials compares performance of the autoencoders with different numbers of latent dimension d and different aggregation strategies. Surprisingly, a simpler averaging aggregation performs better than the RNN aggregation. We attribute it to the small number of training examples in the dataset which seems to be insufficient to train a bidirectional LSTM for aggregation with the state-of-the-art generalization performance.

**Table 2:** Comparison of performance of different AF detection algorithms evaluated on Physionet Challenge data. For our approaches, average F1-score, sensitivity, specificity, PPV, area under the ROC curve (AUC), and accuracy (ACC) of 10-fold cross-validation are presented with the standard deviation. F1 values marked with ∗ are estimated from the corresponding sensitivity and PPV reported in the literature.

Method	F1	Sensitivity	Specificity	PPV	AUC	ACC
DHB+RNN	81.3±3.2	79.9±4.9	97.5±0.6	82.9±3.2	96.3±1.1	95.2±0.7
P-DHB+RNN	75.4±3.6	68.8±5.4	98.0±0.6	83.8±4.0	94.9±0.9	94.2±0.8
S-DHB+RNN	73.9±4.0	64.1±6.3	98.7±0.6	88.1±4.0	94.0±1.1	94.2±0.6
DHB+avg	84.5±3.0	80.2±4.6	98.6±0.4	89.6±3.0	97.3±0.6	96.2±0.7
P-DHB+avg	88.6±2.3	88.2±3.0	98.4±0.6	89.1±3.4	98.3±0.6	97.0±0.6
S-DHB+Avg	82.7±1.9	78.2±4.2	98.4±0.5	88.0±3.0	96.3±1.1	95.8±0.4
MS-CNN [20]	90.5${}^{*}$	89.7	98.8	91.4	—	97.8
AliveCor [20]	—	85.0	90.0	—	—	—
VGGNet [20]	86.57${}^{*}$	87.9	98.1	85.3	—	96.9
CNN [36]	58.4${}^{*}$	59.9	93.4	56.9	—	89.1

To explore further how well our approach generalizes for other datasets, we then have applied our method to solve AF detection tasks using the MIT-BIH Atrial Fibrillation Database (AFDB) [31] and the MIT-BIH Arrhythmia Database (MITDB) [28]. In addition, we conduct these experiments to demonstrate that the proposed encoders learn transferable representations and that they are capable of extracting useful features from ECG signals even when the signals come from another databases and have been collected by different devices in different settings. To achieve this aim, we reuse the DHB, P-DHB, and S-DHB autoencoders trained using the Physionet Challenge data for encoding heartbeats of the new ECG recordings before training the rest of the pipeline for solving the classification tasks.

The MIT-BIH Atrial Fibrillation Database (AFDB) [31] contains 23 (two records were excluded from the consideration as the ECG signal is not available) 2-channel ECG recordings with approximately ten-hour duration at 250 Hz of 12-bit resolution over a range of ±10 mV. The recordings in this database contain mostly atrial fibrillation (AF) and normal sinus rhythm (NSR). The MIT-BIH Arrhythmia Database (MITDB) [28] collects 48 half-hour two-channel ECG recordings that were digitized at 360 Hz with 11-bit resolution over a 10 mV range. Unlike the previous database, the signals represent a variety of rhythms including ventricular bigeminy and trigeminy. Therefore, the MITDB defines a more challenging task than previous comparisons.

Following the studies [23–25], we classify fixed-length windows of consecutive heartbeats of an ECG record into AF and non-AF. To compare our method with the aforementioned studies, we use sliding windows of 30 heartbeats as an input data and annotated the whole window according to a majority of the heartbeats in that window [25, 27]. This pooling strategy means that a window was labeled as AF in case at least 15 out 30 heartbeats in that window have AF annotation. Previous work suggests three main strategies to produce the labels for the whole window of heartbeats: (i) annotation of the heartbeat in the center (middle) [23]; (ii) annotation of the majority of the heartbeats in the windows (majority) [25]; (iii) if the percentage of the AF heartbeats exceeds a threshold, e.g., p=80% (threshold) [24]. Table S7 in Supplementary Materials summarizes discrepancy between the aforementioned labeling strategies and the median of these three annotations. Based on the high agreement between all three strategies, we conclude that the choice of them has low impact on the classification results. We picked the majority labeling because it has the highest overlap with the median consensus of the three proposed strategies.

We like to emphasize that for these experiments the autoencoder models trained on the Physionet Challenge data from the previous step are used to process new data for extracting heartbeat features without any additional fine-tuning of DHB, P-DHB, or S-DHB. Similar to the first experiment, no preprocessing, filtering, or normalization was applied to the recording before encoding. Further, we trained only aggregating networks and classifiers to perform a binary classification AF vs. non-AF on each database separately. Since the Physionet Challenge dataset contains single-lead ECGs, we encoded only the first lead of two-lead ECG signals from AFDB and MITDB databases.

Tables 3 and 4 summarize the comparison of our proposed approaches with state-of-the-art methods. We see that Shape-DeepHeartBeat in combination with the RNN aggregation outperforms other proposed configurations as well as other approaches. Tables S5 and S6 from Supplementary Materials documents the performance of DeepHeartBeat, Pace-DeepHeartBeat, and Shape-DeepHeartBeat with different numbers of latent dimensions d and different aggregation functions. Since the AFDB and MITDB datatsets contain significantly more training samples, the RNN aggregation can train and generalize well and outperforms the averaging. We explain the good performance of Shape-DeepHeartBeat with the better quality of transferred representation. S-DHP parameterisation does not include a pace parameter, and therefore, the S-DHP autoencoder proves to be more robust against changes of sampling frequency than alternative methods. Good performance of Shape-DeepHeartBeat, which does not explicitly store information on the length of cardiac cycles, also suggests that other features might be indicative for atrial fibrillation, although this information only relates to the shape of heartbeats. This finding agrees with the fact that averaging aggregation still yields good results in AF detection as shown in the previous experiment, despite discarding information on the order and variability of heartbeats.

**Table 3:** Comparison of performance of different AF detection algorithms evaluated on the AFDB data. For our approaches, average F1-score, sensitivity, specificity, PPV, area under the ROC curve (AUC), and accuracy (ACC) of 10-fold cross-validation are presented with the standard deviation. 30 heartbeat windows are considered an input. Each window is labeled according to the majority of the heartbeat annotations of the window. F1 values marked with ∗ are estimated from the corresponding sensitivity and PPV reported in the literature.

Method	F1	Sensitivity	Specificity	PPV	AUC	ACC
DHB+RNN	98.5±0.2	98.7±0.3	98.6±0.2	98.3±0.3	99.8±0.0	98.7±0.1
P-DHB+RNN	98.6±0.2	98.7±0.3	98.8±0.1	98.5±0.2	99.8±0.0	98.8±0.2
S-DHB+RNN	98.8±0.1	98.9±0.3	98.9±0.2	98.7±0.2	99.8±0.0	98.9±0.1
DHB+avg	96.6±0.2	97.6±0.4	96.3±0.4	95.6±0.4	99.4±0.1	96.9±0.2
P-DHB+avg	93.7±0.5	95.6±1.2	93.0±1.1	91.8±1.2	97.9±0.2	94.2±0.5
S-DHB+avg	96.8±0.2	97.7±0.4	96.6±0.4	96.0±0.5	99.4±0.1	97.1±0.2
Asgari et al. [27]	—	97.0	97.1	—	—	—
Islam et al. [23]	94.9${}^{*}$	95.8	95.5	94.1	—	95.6
Andersen et al. [25]	97.3${}^{*}$	99.0±0.2	97.0±1.6	95.8±2.7	—	97.8±0.6

**Table 4:** Comparison of performance of different AF detection algorithms evaluated on the MITDB data. For our approaches, average F1-score, sensitivity, specificity, PPV, area under the ROC curve (AUC), and accuracy (ACC) of 10-fold cross-validation are presented with the standard deviation. 30 heartbeat windows are considered an input. Each window is labeled according to the majority of the heartbeat annotations of the window. F1 values marked with ∗ are estimated from the corresponding sensitivity and PPV reported in the literature.

Method	F1	Sensitivity	Specificity	PPV	AUC	ACC
DHB+RNN	93.3±1.7	96.1±1.7	98.9±0.4	90.8±2.8	99.5±0.3	98.6±0.4
P-DHB+RNN	94.6±1.0	97.4±1.9	99.0±0.1	92.1±0.4	99.7±0.2	98.9±0.2
S-DHB+RNN	94.3±1.3	96.1±1.2	99.1±0.2	92.6±1.9	99.6±0.2	98.8±0.3
DHB+avg	71.3±2.8	90.8±3.1	92.6±1.0	58.9±3.5	96.4±0.6	92.4±0.9
P-DHB+avg	76.1±2.9	88.7±4.7	94.8±1.2	67.0±4.8	97.8±0.5	94.2±0.9
S-DHB+avg	72.4±2.0	85.5±7.1	94.1±1.2	63.3±3.1	96.9±0.8	93.2±0.5
Islam et al. [23]	56.0${}^{*}$	86.2	86.0	41.5	—	86.0
Andersen et al. [25]	62.3${}^{*}$	99.0	86.0	45.5	—	87.4

## 5. Discussion

Our research program provides a three-step pipeline for processing ECG signals and for detecting atrial fibrillation (AF). First, the method splits the input recording into a sequence of individual heart cycles for extracting heartbeat features with a DeepHeartBeat-type encoder. Second, these learnt encodings are aggregated to capture the heart dynamics. This decomposition in heartbeat features and the heartbeat rhythm allows us to study the signal on two levels and, thereby, takes into consideration the shape features, the duration of individual heartbeats, and the heart rhythm of the entire signal. This design choice reflects the known observation that AF can show itself both as rhythm irregularity and as abnormal heartbeat shape, e.g., absence of the P-wave or changes in the QRS-complex.

Our approach shows over 90% classification accuracy on the task of detecting AF from a single-lead noisy ECG recording on all three considered datasets: Physionet Challenge, MIT-BIH Atrial Fibrillation (AFDB), MIT-BIH Arrhythmia (MITDB) databases. This performance exceeds the detection rate of existing ECG processing algorithms. Furthermore, we have observed a statistical dependence between atrial fibrillation events and indicative heartbeat shape features. These features might complement information about heart rhythm and heart rate which is definitely relevant for detection of atrial fibrillation.

High performance of our method on unseen AFDB and MITDB databases that were not used to train the autoencoders confirms that autoencoders are able to produce transferable representations that generalize on signals acquired by different machines with different settings and sampling rates. We also like to emphasize that all three different DeepHeartBeat-like autoencoders exhibit their specific, setting-dependent advantages and can be beneficially applied there. For example, unlike other two, DeepHeartBeat does not require the positions of R-peaks and hence it is not affected by possible errors in R-peak detection. Likewise, Shape-DeepHeartBeat is highly robust to changes in signal frequency and, therefore, exhibits excellent transferability between data sources since it discards the information about heartbeat duration and sampling frequency. Finally, Pace-DeepHeartBeat excels with the best performance as it utilizes all signal information necessary for AF classification and discards irrelevant features such as shift parameters.

Although we mostly considered the AF detection as a downstream task in this work, we would like to emphasize that the ECG features extracted by the proposed autoencoders are not specific or tailored for this task. The autoencoders are trained in an unsupervised manner to reconstruct the signal and do not consider any labels for this process. Therefore, the extracted features may prove to be useful for other tasks including heartbeat classification or diagnosis of other cardiovascular diseases. Only the aggregation and classification part of our pipeline should be retrained for new tasks in contrast to other approaches that train the feature extractor jointly with the classifier.

The presented work, however, still exposes some limitations for explaining the detection process. While the learnt DHB-type embeddings model the heart cycle explicitly and, hence, provide interpretations of the embeddings; the subsequent parts of the pipeline, such as RNN aggregation, still lack interpretability, not to mention a causal analysis. Employing attention [8] for the aggregating RNN model as suggested in [37, 38] could provide insights which parts of the input signal prove most relevant for influencing a classification decision. In general, a further study of heart rhythm and dynamics of the heartbeat embeddings appears as a promising direction to interpret such dependencies. The good performance of models that discard information on the heart rate and the order of heartbeats also supports the hypothesis that our deep learning architecture effectively classifies heart rhythm patterns by filtering out rhythm-relevant information from ECG data.

## Data Availability

All the data for the experiments is publicly available via the PhysioNet databank [35]. The Physionet Challenge data was downloaded from the official page of the PhysioNet/Computing in Cardiology Challenge 2017 (https://physionet.org/content/challenge-2017/1.0.0/). The AFDB data was downloaded from the official page of the MIT-BIH Attrial Fibrillation Database (https://physionet.org/content/afdb/1.0.0/). The MITDB data was downloaded from the official page of the MIT-BIH Arrhythmia Database (https://physionet.org/content/mitdb/1.0.0/).

## Additional Points

*Code Availability*. The code for experiments and visualisations from this paper is available on GitHub via https://github.com/alinadubatovka/DeepHeartBeat-ECG.

## Supplementary Materials

Supplementary Materials Table S1: summary of the Physionet Challenge dataset. Supplementary methods: neural network architecture. Table S2: neural network architecture of the aggregating RNN. The size of the input depends on the number of latent dimensions of the encoder (d=8, 16, 32, and 64). m denotes the number of heartbeats or slices representing an ECG recording. Table S3: neural network architecture of the classification head (MLP). The size of the input depends on the dimensionality of the signal’s embedding. The final output has dimensionality 4 equal to the number of classes. Note that the classifier outputs logits instead of normalized probabilities. Figure S1: reconstructions of different heartbeats from the same patient produced by DeepHeartBeat, Pace-DeepHeartBeat, and Shape-DeepHeartBeat with different numbers of latent dimensions d. Table S4: performance of different versions of DeepHeartBeat on AF detection with averaging and RNN as an aggregation on the Physionet Challenge dataset. Average F1-score, sensitivity, specificity, PPV, area under the ROC curve (AUC), and accuracy (ACC) of 10-fold cross-validation are presented with the standard deviation. Table S5: performance of different versions of DeepHeartBeat on AF detection with averaging and RNN as an aggregation on the AFDB database. Average F1-score, sensitivity, specificity, PPV, area under the ROC curve (AUC), and accuracy (ACC) of 10-fold cross-validation are presented with the standard deviation. 30 heartbeat windows are considered an input. Each window is labeled according to the majority of the heartbeat annotations of the window. Table S6: performance of different versions of DeepHeartBeat on AF detection with averaging and RNN as an aggregation on the MITDB database. Average F1-score, sensitivity, specificity, PPV, area under the ROC curve (AUC), and accuracy (ACC) of 10-fold cross-validation are presented with the standard deviation. 30 heartbeat windows are considered an input. Each window is labeled according to the majority of the heartbeat annotations of the window. Table S7: agreement between different labeling strategies and their consensuses for the AFDB database. *All* corresponds to the median of all three strategies; *rest* stands for the median consensus between two other strategies (e.g., for the *majority* strategy, *rest* consensus corresponds to the median between *majority* and *threshold*).
