# Supplementary material for: Automatic Detection of Atrial Fibrillation from Single-Lead ECG Using Deep Learning of the Cardiac Cycle
Source: BME Front. 2022 Apr 12;2022:9813062. doi: 10.34133/2022/9813062 (PMC10521743; doi:10.34133/2022/9813062)
Supplement: Supplementary Materials — Table S1: summary of the Physionet Challenge dataset. Supplementary methods: neural network architecture. Table S2: neural network architecture of the aggregating RNN. The size of the input depends on the number of latent dimensions of the encoder (d=8, 16, 32, and 64). m denotes the number of heartbeats or slices representing an ECG recording. Table S3: neural network architecture of the classification head (MLP). The size of the input depends on the dimensionality of the signal’s embedding. The final output has dimensionality 4 equal to the number of classes. Note that the classifier outputs logits instead of normalized probabilities. Figure S1: reconstructions of different heartbeats from the same patient produced by DeepHeartBeat, Pace-DeepHeartBeat, and Shape-DeepHeartBeat with different numbers of latent dimensions d. Table S4: performance of different versions of DeepHeartBeat on AF detection with averaging and RNN as an aggregation on the Physionet Challenge dataset. Average F1-score, sensitivity, specificity, PPV, area under the ROC curve (AUC), and accuracy (ACC) of 10-fold cross-validation are presented with the standard deviation. Table S5: performance of different versions of DeepHeartBeat on AF detection with averaging and RNN as an aggregation on the AFDB database. Average F1-score, sensitivity, specificity, PPV, area under the ROC curve (AUC), and accuracy (ACC) of 10-fold cross-validation are presented with the standard deviation. 30 heartbeat windows are considered an input. Each window is labeled according to the majority of the heartbeat annotations of the window. Table S6: performance of different versions of DeepHeartBeat on AF detection with averaging and RNN as an aggregation on the MITDB database. Average F1-score, sensitivity, specificity, PPV, area under the ROC curve (AUC), and accuracy (ACC) of 10-fold cross-validation are presented with the standard deviation. 30 heartbeat windows are considered an input. Each window is labeled accordin [file 9813062.f1.pdf]

# Automatic Detection of Atrial Fibrillation from Single-lead ECG Using Deep Learning of Cardiac Cycle

Alina Dubatovka[1*] and Joachim M. Buhmann[1]

[1]Department of Computer Science, ETH Zurich, Zurich, Switzerland.
[*]Corresponding author. Email: alina.dubatovka@inf.ethz.ch

## Supplementary Materials

Table S1: Summary of the Physionet dataset.

| Class | Count | Mean (s) | Std (s) | Min (s) | Max (s) |
|---|---|---|---|---|---|
| Normal sinus rhythm (NSR) | 5154 | 31.9 | 10.0 | 9.0 | 61.0 |
| Atrial Fibrillation (AF) | 771 | 31.6 | 12.5 | 10.0 | 60.0 |
| Other rhythms (O) | 2557 | 34.1 | 11.8 | 9.1 | 60.9 |
| Noise (N) | 46 | 27.1 | 9 | 10.2 | 60.0 |
| Total | 8528 | 32.5 | 10.9 | 9.0 | 61.0 |

## Supplementary methods

### Neural network architecture

For training DeepHeartBeat, we used the same architecture and training procedure as was originally proposed in [1]. For Pace-DeepHeartBeat and Shape-DeepHeartBeat, however, we modified the observation encoder network part of the DeepHeartBeat encoder to make it output $d-1$ and $d-2$ parameters respectively. The original DHB trajectory outlined in Equation 1 was then replaced by the trajectories presented by Equations 2 and 3. Because Shape-DeepHeartBeat does not model frequency, we did not use a heart beat regularisation term as for DeepHeartBeat and Pace-DeepHeartBeat.

Table S2: Neural network architecture of the aggregating RNN. The size of the input depends on the number of latent dimensions of the encoder ($d = 8,\ 16,\ 32,\ 64$). $m$ denotes the number of heart beats or slices representing an ECG recording.

| Layer type. Number of units. Activation function. | Output shape |
|---|---|
| Input (sequence of heart beat embeddings). | $m \times d$ |
| Bidirectional LSTM. 64 units per direction. Tanh. | 128 |

Table S3: Neural network architecture of the classification head (MLP). The size of the input depends on the dimensionality of the signal's embedding. The final output has dimensionality 4 equal to the number of classes. Note that the classifier outputs logits instead of normalized probabilities.

| Layer type. Number of units. Activation function. | Output shape |
| --- | --- |
| Input (embedding $\varphi_i$ of the entire signal). | $d$ |
| Fully-connected. 64 units. ReLU. | 64 |
| Fully-connected. 16 units. ReLU. | 16 |
| Fully-connected. 4 units. ReLU. | 4 |

We trained the classifier jointly with the aggregation neural network using Adam optimiser [2] with learning rate 0.001. The exponential decay rates were set to default values $\beta_1 = 0.9$ for the first and $\beta_2 = 0.999$ for the second moment estimates. We run 100 epoch iterating over training dataset and use batch size of 64 signal embeddings. To speed up and stabilise training, we initialised weights of all fully-connected layers of the classifier using the orthogonal initialization proposed in [3]. The categorical cross-entropy was used as the loss function for training the classifier. To alleviate class imbalance in the data, we re-sampled training dataset to equalise number of samples from each class exposed to a model during training. The validation dataset was left untouched. All classification results presented in the paper are calculated for original class-imbalanced data.

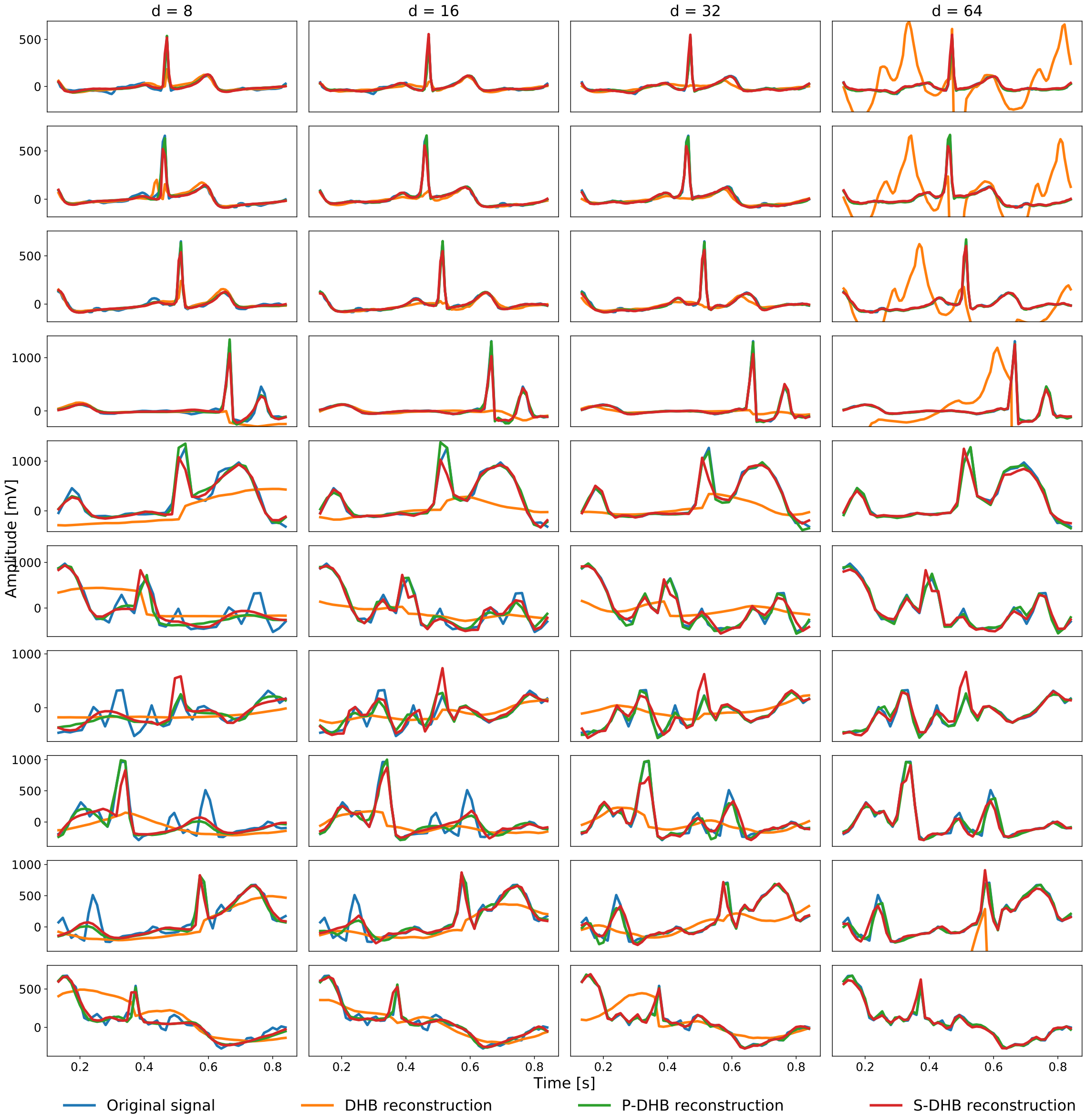


Figure S1: Reconstructions of different heart beats from the same patient produced by DeepHeartBeat, Pace-DeepHeartBeat, and Shape-DeepHeartBeat with different number of latent dimensions $d$.

Table S4: Performance of different versions of DeepHeartBeat on AF detection with averaging and RNN as an aggregation on the Physionet Challenge dataset. Average $F_1$-score, Sensitivity, Specificity, PPV, area under the ROC curve (AUC), and accuracy (ACC) of 10-fold cross-validation are presented with the standard deviation.

| Encoder | Dims | $F_1$ | Sensitivity | Specificity | PPV | AUC | ACC |
|---|---|---|---|---|---|---|---|
| **RNN** | | | | | | | |
| DHB | d = 8 | $46.1 \pm 7.2$ | $32.2 \pm 7.6$ | $99.1 \pm 0.6$ | $85.5 \pm 6.9$ | $81.5 \pm 3.1$ | $90.4 \pm 0.7$ |
| | d = 16 | $46.0 \pm 5.3$ | $32.2 \pm 4.9$ | $98.9 \pm 0.6$ | $82.6 \pm 8.5$ | $79.9 \pm 3.4$ | $90.2 \pm 0.7$ |
| | d = 32 | $60.4 \pm 5.4$ | $48.9 \pm 7.8$ | $98.2 \pm 1.2$ | $81.9 \pm 8.0$ | $89.0 \pm 1.8$ | $91.8 \pm 0.7$ |
| | d = 64 | $81.3 \pm 3.2$ | $79.9 \pm 4.9$ | $97.5 \pm 0.6$ | $82.9 \pm 3.2$ | $96.3 \pm 1.1$ | $95.2 \pm 0.7$ |
| P-DHB | d = 8 | $53.6 \pm 4.7$ | $38.6 \pm 5.4$ | $99.3 \pm 0.5$ | $89.8 \pm 5.1$ | $82.9 \pm 3.5$ | $91.4 \pm 0.5$ |
| | d = 16 | $72.5 \pm 4.1$ | $65.4 \pm 6.7$ | $97.8 \pm 0.8$ | $82.0 \pm 4.2$ | $93.0 \pm 2.0$ | $93.6 \pm 0.8$ |
| | d = 32 | $72.6 \pm 2.8$ | $64.1 \pm 4.1$ | $98.2 \pm 0.7$ | $84.3 \pm 5.0$ | $94.1 \pm 1.0$ | $93.7 \pm 0.6$ |
| | d = 64 | $75.4 \pm 3.6$ | $68.8 \pm 5.4$ | $98.0 \pm 0.6$ | $83.8 \pm 4.0$ | $94.9 \pm 0.9$ | $94.2 \pm 0.8$ |
| S-DHB | d = 8 | $51.5 \pm 5.2$ | $37.0 \pm 5.4$ | $99.1 \pm 0.4$ | $86.4 \pm 5.2$ | $83.9 \pm 2.4$ | $91.0 \pm 0.6$ |
| | d = 16 | $69.4 \pm 4.6$ | $59.8 \pm 6.2$ | $98.2 \pm 0.6$ | $83.4 \pm 5.0$ | $92.9 \pm 1.4$ | $93.2 \pm 0.9$ |
| | d = 32 | $73.7 \pm 3.7$ | $67.2 \pm 5.0$ | $97.8 \pm 0.6$ | $81.8 \pm 4.1$ | $94.0 \pm 1.2$ | $93.8 \pm 0.8$ |
| | d = 64 | $73.9 \pm 4.0$ | $64.1 \pm 6.3$ | $98.7 \pm 0.6$ | $88.1 \pm 4.0$ | $94.0 \pm 1.1$ | $94.2 \pm 0.6$ |
| **Averaging** | | | | | | | |
| DHB | d = 8 | $54.8 \pm 8.3$ | $42.1 \pm 9.2$ | $98.5 \pm 0.6$ | $81.3 \pm 5.0$ | $85.2 \pm 3.5$ | $91.2 \pm 1.0$ |
| | d = 16 | $61.1 \pm 6.5$ | $49.5 \pm 7.4$ | $98.2 \pm 0.9$ | $81.2 \pm 7.8$ | $88.3 \pm 2.0$ | $91.9 \pm 1.1$ |
| | d = 32 | $72.2 \pm 2.9$ | $62.7 \pm 3.7$ | $98.4 \pm 0.4$ | $85.3 \pm 2.9$ | $92.8 \pm 2.0$ | $93.7 \pm 0.6$ |
| | d = 64 | $84.5 \pm 3.0$ | $80.2 \pm 4.6$ | $98.6 \pm 0.4$ | $89.6 \pm 3.0$ | $97.3 \pm 0.6$ | $96.2 \pm 0.7$ |
| P-DHB | d = 8 | $50.8 \pm 4.4$ | $35.4 \pm 4.2$ | $99.5 \pm 0.4$ | $91.5 \pm 6.8$ | $81.8 \pm 3.1$ | $91.1 \pm 0.6$ |
| | d = 16 | $75.5 \pm 6.0$ | $68.5 \pm 9.7$ | $98.2 \pm 0.6$ | $85.4 \pm 3.0$ | $94.4 \pm 2.0$ | $94.4 \pm 0.9$ |
| | d = 32 | $81.5 \pm 3.7$ | $76.2 \pm 4.9$ | $98.4 \pm 0.5$ | $87.8 \pm 3.7$ | $96.6 \pm 0.9$ | $95.5 \pm 0.9$ |
| | d = 64 | $88.6 \pm 2.3$ | $88.2 \pm 3.0$ | $98.4 \pm 0.6$ | $89.1 \pm 3.4$ | $98.3 \pm 0.6$ | $97.0 \pm 0.6$ |
| S-DHB | d = 8 | $55.3 \pm 6.0$ | $43.3 \pm 7.3$ | $98.2 \pm 0.6$ | $78.5 \pm 3.6$ | $84.9 \pm 2.4$ | $91.1 \pm 0.6$ |
| | d = 16 | $75.1 \pm 3.0$ | $69.9 \pm 6.0$ | $97.6 \pm 1.0$ | $81.9 \pm 5.8$ | $94.5 \pm 0.7$ | $94.0 \pm 0.7$ |
| | d = 32 | $80.9 \pm 2.8$ | $76.5 \pm 4.5$ | $98.1 \pm 0.5$ | $86.1 \pm 3.4$ | $96.1 \pm 0.9$ | $95.3 \pm 0.6$ |
| | d = 64 | $82.7 \pm 1.9$ | $78.2 \pm 4.2$ | $98.4 \pm 0.5$ | $88.0 \pm 3.0$ | $96.3 \pm 1.1$ | $95.8 \pm 0.4$ |

Table S5: Performance of different versions of DeepHeartBeat on AF detection with averaging and RNN as an aggregation on the AFDB database. Average $F_1$-score, Sensitivity, Specificity, PPV, area under the ROC curve (AUC), and accuracy (ACC) of 10-fold cross-validation are presented with the standard deviation. 30 heart beats windows are considered as an input. Each window is labeled according to the majority of the heart beat annotations of the window.

| Encoder | Dims | $F_1$ | Sensitivity | Specificity | PPV | AUC | ACC |
|---|---|---|---|---|---|---|---|
| | | | **RNN** | | | | |
| DHB | d = 8 | $98.5 \pm 0.2$ | $98.7 \pm 0.3$ | $98.6 \pm 0.2$ | $98.3 \pm 0.3$ | $99.8 \pm 0.0$ | $98.7 \pm 0.1$ |
| | d = 16 | $98.7 \pm 0.2$ | $98.9 \pm 0.5$ | $98.7 \pm 0.3$ | $98.5 \pm 0.3$ | $99.8 \pm 0.0$ | $98.8 \pm 0.2$ |
| | d = 32 | $98.5 \pm 0.4$ | $99.0 \pm 0.2$ | $98.4 \pm 0.6$ | $98.1 \pm 0.7$ | $99.8 \pm 0.1$ | $98.7 \pm 0.4$ |
| | d = 64 | $98.3 \pm 0.1$ | $98.5 \pm 0.3$ | $98.4 \pm 0.3$ | $98.1 \pm 0.3$ | $99.7 \pm 0.0$ | $98.5 \pm 0.1$ |
| P-DHB | d = 8 | $98.6 \pm 0.1$ | $98.8 \pm 0.2$ | $98.7 \pm 0.2$ | $98.4 \pm 0.3$ | $99.8 \pm 0.0$ | $98.7 \pm 0.1$ |
| | d = 16 | $98.5 \pm 0.2$ | $98.8 \pm 0.3$ | $98.6 \pm 0.4$ | $98.3 \pm 0.4$ | $99.8 \pm 0.0$ | $98.7 \pm 0.2$ |
| | d = 32 | $98.6 \pm 0.2$ | $98.7 \pm 0.3$ | $98.8 \pm 0.1$ | $98.5 \pm 0.2$ | $99.8 \pm 0.0$ | $98.8 \pm 0.2$ |
| | d = 64 | $98.5 \pm 0.1$ | $98.8 \pm 0.3$ | $98.5 \pm 0.2$ | $98.2 \pm 0.2$ | $99.8 \pm 0.0$ | $98.6 \pm 0.1$ |
| S-DHB | d = 8 | $98.7 \pm 0.1$ | $98.8 \pm 0.2$ | $98.8 \pm 0.2$ | $98.6 \pm 0.3$ | $99.8 \pm 0.0$ | $98.8 \pm 0.1$ |
| | d = 16 | $98.8 \pm 0.1$ | $98.9 \pm 0.3$ | $98.9 \pm 0.2$ | $98.7 \pm 0.2$ | $99.8 \pm 0.0$ | $98.9 \pm 0.1$ |
| | d = 32 | $98.7 \pm 0.1$ | $99.0 \pm 0.1$ | $98.7 \pm 0.2$ | $98.4 \pm 0.2$ | $99.8 \pm 0.0$ | $98.8 \pm 0.1$ |
| | d = 64 | $98.7 \pm 0.1$ | $98.9 \pm 0.2$ | $98.8 \pm 0.2$ | $98.5 \pm 0.3$ | $99.8 \pm 0.0$ | $98.8 \pm 0.1$ |
| | | | **Averaging** | | | | |
| DHB | d = 8 | $95.3 \pm 0.2$ | $96.8 \pm 0.5$ | $94.7 \pm 0.6$ | $93.8 \pm 0.6$ | $98.9 \pm 0.0$ | $95.7 \pm 0.2$ |
| | d = 16 | $96.5 \pm 0.2$ | $97.8 \pm 0.5$ | $96.1 \pm 0.2$ | $95.3 \pm 0.2$ | $99.3 \pm 0.0$ | $96.8 \pm 0.2$ |
| | d = 32 | $96.6 \pm 0.2$ | $97.6 \pm 0.4$ | $96.3 \pm 0.4$ | $95.6 \pm 0.4$ | $99.4 \pm 0.1$ | $96.9 \pm 0.2$ |
| | d = 64 | $97.1 \pm 0.2$ | $98.0 \pm 0.4$ | $96.8 \pm 0.3$ | $96.2 \pm 0.3$ | $99.5 \pm 0.1$ | $97.4 \pm 0.2$ |
| P-DHB | d = 8 | $84.4 \pm 3.3$ | $90.2 \pm 5.0$ | $80.7 \pm 5.7$ | $79.6 \pm 4.3$ | $90.9 \pm 2.7$ | $85.0 \pm 3.4$ |
| | d = 16 | $85.9 \pm 0.5$ | $89.8 \pm 1.7$ | $84.2 \pm 1.2$ | $82.3 \pm 0.9$ | $93.7 \pm 0.3$ | $86.7 \pm 0.4$ |
| | d = 32 | $91.9 \pm 0.5$ | $97.2 \pm 0.7$ | $88.3 \pm 1.0$ | $87.2 \pm 1.0$ | $96.5 \pm 0.6$ | $92.3 \pm 0.5$ |
| | d = 64 | $93.7 \pm 0.5$ | $95.6 \pm 1.2$ | $93.0 \pm 1.1$ | $91.8 \pm 1.2$ | $97.9 \pm 0.2$ | $94.2 \pm 0.5$ |
| S-DHB | d = 8 | $95.4 \pm 0.3$ | $96.7 \pm 0.5$ | $95.0 \pm 0.5$ | $94.1 \pm 0.5$ | $99.0 \pm 0.1$ | $95.8 \pm 0.2$ |
| | d = 16 | $96.7 \pm 0.2$ | $97.7 \pm 0.5$ | $96.5 \pm 0.3$ | $95.8 \pm 0.3$ | $99.4 \pm 0.0$ | $97.0 \pm 0.2$ |
| | d = 32 | $96.2 \pm 0.2$ | $97.5 \pm 0.3$ | $95.7 \pm 0.4$ | $94.9 \pm 0.5$ | $99.2 \pm 0.1$ | $96.5 \pm 0.2$ |
| | d = 64 | $96.8 \pm 0.2$ | $97.7 \pm 0.4$ | $96.6 \pm 0.4$ | $96.0 \pm 0.5$ | $99.4 \pm 0.1$ | $97.1 \pm 0.2$ |

Table S6: Performance of different versions of DeepHeartBeat on AF detection with averaging and RNN as an aggregation on the MITDB database. Average $F_1$-score, Sensitivity, Specificity, PPV, area under the ROC curve (AUC), and accuracy (ACC) of 10-fold cross-validation are presented with the standard deviation. 30 heart beats windows are considered as an input. Each window is labeled according to the majority of the heart beat annotations of the window.

| Encoder | Dims | $F_1$ | Sensitivity | Specificity | PPV | AUC | ACC |
|---|---|---|---|---|---|---|---|
| **RNN** | | | | | | | |
| DHB | d = 8 | $91.3 \pm 3.08$ | $95.2 \pm 0.4$ | $98.4 \pm 0.8$ | $87.9 \pm 5.3$ | $99.4 \pm 0.3$ | $98.1 \pm 0.7$ |
| | d = 16 | $91.7 \pm 2.1$ | $96.9 \pm 1.3$ | $98.3 \pm 0.5$ | $87.1 \pm 3.5$ | $99.5 \pm 0.2$ | $98.1 \pm 0.5$ |
| | d = 32 | $91.9 \pm 1.4$ | $94.5 \pm 1.5$ | $98.7 \pm 0.2$ | $89.5 \pm 1.4$ | $99.0 \pm 0.9$ | $98.3 \pm 0.3$ |
| | d = 64 | $93.3 \pm 1.7$ | $96.1 \pm 1.7$ | $989 \pm 0.4$ | $90.8 \pm 2.8$ | $99.5 \pm 0.3$ | $98.6 \pm 0.4$ |
| P-DHB | d = 8 | $91.6 \pm 2.5$ | $96.8 \pm 1.1$ | $98.3 \pm 0.7$ | $86.9 \pm 4.2$ | $99.5 \pm 0.2$ | $98.1 \pm 0.6$ |
| | d = 16 | $93.6 \pm 0.8$ | $97.1 \pm 1.8$ | $98.8 \pm 0.2$ | $90.4 \pm 1.6$ | $99.7 \pm 0.1$ | $98.6 \pm 0.2$ |
| | d = 32 | $94.6 \pm 1.0$ | $97.4 \pm 1.9$ | $99.0 \pm 0.1$ | $92.1 \pm 0.4$ | $99.7 \pm 0.2$ | $98.9 \pm 0.2$ |
| | d = 64 | $94.0 \pm 1.6$ | $96.9 \pm 1.2$ | $98.9 \pm 0.4$ | $91.3 \pm 2.8$ | $99.7 \pm 0.3$ | $98.7 \pm 0.4$ |
| S-DHB | d = 8 | $91.8 \pm 2.9$ | $94.8 \pm 3.5$ | $98.6 \pm 0.6$ | $89.1 \pm 4.2$ | $99.4 \pm 0.3$ | $98.2 \pm 0.7$ |
| | d = 16 | $91.9 \pm 1.4$ | $94.5 \pm 1.5$ | $98.7 \pm 0.2$ | $89.5 \pm 1.4$ | $99.0 \pm 0.9$ | $98.2 \pm 0.3$ |
| | d = 32 | $94.3 \pm 1.3$ | $96.1 \pm 1.2$ | $99.1 \pm 0.2$ | $92.6 \pm 1.9$ | $99.6 \pm 0.2$ | $98.8 \pm 0.3$ |
| | d = 64 | $92.4 \pm 1.8$ | $95.7 \pm 1.8$ | $98.7 \pm 0.5$ | $89.3 \pm 3.2$ | $99.4 \pm 0.3$ | $98.4 \pm 0.4$ |
| **Averaging** | | | | | | | |
| DHB | d = 8 | $55.7 \pm 7.3$ | $82.8 \pm 6.9$ | $85.7 \pm 8.0$ | $42.7 \pm 7.1$ | $91.5 \pm 4.6$ | $85.4 \pm 6.9$ |
| | d = 16 | $57.7 \pm 3.0$ | $70.2 \pm 7.2$ | $91.5 \pm 1.4$ | $49.2 \pm 3.0$ | $92.1 \pm 1.5$ | $89.3 \pm 0.9$ |
| | d = 32 | $62.1 \pm 7.1$ | $84.8 \pm 9.9$ | $89.7 \pm 2.3$ | $49.2 \pm 6.4$ | $94.1 \pm 3.0$ | $89.2 \pm 2.4$ |
| | d = 64 | $71.3 \pm 2.8$ | $90.8 \pm 3.1$ | $92.6 \pm 1.0$ | $58.9 \pm 3.5$ | $96.4 \pm 0.6$ | $92.4 \pm 0.9$ |
| P-DHB | d = 8 | $61.9 \pm 1.6$ | $84.0 \pm 3.7$ | $89.8 \pm 1.3$ | $49.2 \pm 2.4$ | $94.7 \pm 0.4$ | $89.2 \pm 0.9$ |
| | d = 16 | $70.2 \pm 2.1$ | $89.7 \pm 3.7$ | $92.3 \pm 1.2$ | $57.8 \pm 3.1$ | $97.1 \pm 0.4$ | $92.0 \pm 0.9$ |
| | d = 32 | $76.1 \pm 2.9$ | $88.7 \pm 4.7$ | $94.8 \pm 1.2$ | $67.0 \pm 4.8$ | $97.8 \pm 0.5$ | $94.2 \pm 0.9$ |
| | d = 64 | $73.4 \pm 1.4$ | $91.2 \pm 1.9$ | $93.3 \pm 0.8$ | $61.5 \pm 2.4$ | $97.7 \pm 0.2$ | $93.1 \pm 0.6$ |
| S-DHB | d = 8 | $56.1 \pm 2.7$ | $79.3 \pm 6.4$ | $88.0 \pm 0.7$ | $43.5 \pm 15$ | $92.0 \pm 1.2$ | $87.1 \pm 0.5$ |
| | d = 16 | $57.7 \pm 4.9$ | $76.6 \pm 13.7$ | $89.8 \pm 2.2$ | $46.9 \pm 3.2$ | $92.2 \pm 3.3$ | $88.4 \pm 1.2$ |
| | d = 32 | $72.4 \pm 2.0$ | $85.5 \pm 7.1$ | $94.1 \pm 1.2$ | $63.3 \pm 3.1$ | $96.9 \pm 0.8$ | $93.2 \pm 0.5$ |
| | d = 64 | $67.5 \pm 2.2$ | $87.8 \pm 4.2$ | $91.6 \pm 1.0$ | $54.9 \pm 2.5$ | $96.1 \pm 0.7$ | $91.2 \pm 0.8$ |

Table S7: Agreement between different labeling strategies and their consensuses for AFDB database. *All* corresponds to the median of all three strategies; *rest* stands for the median consensus between two other strategies (e.g. for *majority* strategy *rest* consensus corresponds to the median between *majority* and *threshold*).

| Labels | Consensus | $F_1$ | Sensitivity | Specificity | PPV | ROC AUC | ACC |
|---|---|---|---|---|---|---|---|
| middle | rest | 99.5 | 100.0 | 99.3 | 99.0 | 99.6 | 99.6 |
| | all | 99.9 | 100.0 | 99.9 | 99.9 | 100.0 | 99.9 |
| majority | rest | 99.5 | 100.0 | 99.3 | 99.1 | 99.7 | 99.6 |
| | all | 100.0 | 100.0 | 100.0 | 100.0 | 100.0 | 100.0 |
| threshold | rest | 99.6 | 99.1 | 100.0 | 100.0 | 99.6 | 99.6 |
| | all | 99.6 | 99.1 | 100.0 | 100.0 | 99.6 | 99.6 |

# References


[1] F. Laumer, G. Fringeli, A. Dubatovka, L. Manduchi, and J. M. Buhmann, "Deepheartbeat: Latent trajectory learning of cardiac cycles using cardiac ultrasounds," in *Proceedings of the Machine Learning for Health NeurIPS Workshop*, E. Alsentzer, M. B. A. McDermott, F. Falck, S. K. Sarkar, S. Roy, and S. L. Hyland, Eds., ser. Proceedings of Machine Learning Research, vol. 136, PMLR, Dec. 2020, pp. 194–212. [Online]. Available: `https://proceedings.mlr.press/v136/laumer20a.html`.

[2] D. P. Kingma and J. Ba, "Adam: A method for stochastic optimization," *arXiv preprint arXiv:1412.6980*, 2014.

[3] R. Burkholz and A. Dubatovka, "Initialization of relus for dynamical isometry," *Advances in Neural Information Processing Systems*, vol. 32, pp. 2385–2395, 2019.
